# The Spatial Differentiation and Driving Forces of Ecological Welfare Performance in the Yangtze River Economic Belt

**DOI:** 10.3390/ijerph192214801

**Published:** 2022-11-10

**Authors:** Ling Bai, Tianran Guo, Wei Xu, Kang Luo

**Affiliations:** 1 School of Economics and Management, Nanchang University, Nanchang 330031, China; 2 Department of Geography and Environment, University of Lethbridge, 4401 University Drive West, Lethbridge, AB T1K 3M4, Canada

**Keywords:** ecological welfare performance, spatial differentiation, spatial convergence, Yangtze River Economic Belt

## Abstract

Ecological welfare performance contributes directly to human well-being and regional sustainable development. Improving the regional ecological welfare performance in the process of pursuing green and sustainable development demands theoretical innovation and empirical exploration. Based on the super-efficiency SBM model, this study evaluated the ecological welfare performance of 108 cities during the period of 2009 to 2019. The Dagum Gini coefficient decomposition and spatial convergence model were employed to analyze the differences in ecological welfare performance across and within the study area and explore the underlining causes of such spatial differentiation in the Yangtze River Economic Belt and the upper, middle and lower reaches. It can be seen from the results that: (1) the overall difference in the ecological welfare performance of the Yangtze River Economic Belt is associated with a fluctuating downward trend during the study period. Regional and inter-regional differences were revealed and hypervariable density was identified as the main source of the differences. (2) The ecological welfare performance of the Yangtze River Economic Belt has absolute and conditional *β* convergence, and the ecological welfare performance of each city-region and surrounding urban areas has a positive impact on each other. (3) The difference in the spatial-temporal differentiation trend is manifested by the difference in the convergence rate. The cities in the middle reaches of the Yangtze River have the fastest convergence rate, followed by the cities in the upper reaches, and the cities in the lower reaches are the slowest. This geographic difference is mainly driven by the combined effects of industrial structure, urban characteristics, environmental regulation, foreign direct investment, and transportation accessibility. Finally, it is proposed that future policies should focus on the imbalanced regional development in the study area, and each region needs to explore ways to improve local ecological welfare performance according to local conditions, and ultimately promote the overall green, coordinated and high-quality development in the Yangtze River Economic Belt.

## 1. Introduction

Coupled with the acceleration of urbanization and industrialization, the rapid economic and social development has also brought about a series of ecological and environmental problems in China, such as environmental pollution and resource shortages [1,2], seriously reducing the quality of life and happiness of residents and thus restricting the high-quality economic and social development [3]. In the report of the 19th National Congress of the Communist Party of China, it was proposed that “We will continue to ensure and improve people’s well-being through development; improving people’s livelihood and well-being is the fundamental purpose of development”. (https://www.12371.cn/2017/10/27/ARTI1509103656574313.shtml (accessed on 23 September 2022)). This statement means that the previously adopted two-dimensional concept of sustainable development as economic growth and green ecology needs to be broadened to include welfare enhancement as a third dimension of sustainable development strategy in China. Accordingly, in order to promote regional sustainable development, there is a need to establish a comprehensive regional performance evaluation framework that simultaneously considers economic, social and environmental dimensions under the conditions of resource constraints, environmental protection requirements, and the improvement of people’s well-being. 

Ecological efficiency is a widely adopted index for sustainable development evaluation, but it focuses only on reducing environmental pollution and maximizing regional economic output [4]. Ecological welfare performance (EWP) index is different because it includes social, economic, environmental dimensions with an emphasis on maximizing human well-being with a minimum amount of ecological resource consumption [5]. Furthermore, because it estimates the efficiency of converting ecological input into social welfare output, EWP index addresses the issues of efficiency and fairness emphasized by green economic development and, consequently, the quality of economy growth [6]. Therefore, the EWP index has the potential to be used to evaluate human well-being and sustainable regional development. However, the applicability of the current EWP index remains problematic. First, there is no agreeable indicators that constitute each dimension of the current EWP index. Secondly, how to empirically measure EWP index indicators is uncertain. Third, there are challenges in the existing data collection and analysis methods in implementing the EWP index for sustainable regional development evaluation. This study attempted to tackle these deficiencies in the literature to accelerate to green and sustainable economic development and to achieve the coordinated development of economy, environment and people’s well-being.

As a strategically important region for the national economic development in the new era and a driving force for high-quality development, the Yangtze River Economic Belt (YREB) contributes to more than forty percent of China’s population and GDP with merely 21.4% of the nation’s land. Due to cumulative environmental effects of long-term high-intensity progress and a lack of sustainable development measures and controls, the YREB is faced with pressing social, economic and environmental consequences in recent years including uncoordinated economic development, lagging environmental protection, severe smog pollution and water eutrophication [7]. For instance, its wastewater discharge already accounts for more than 40% of the national discharge. Its unit emission intensity of sulfur dioxide and ammonia nitrogen is more than 1.5 times of the national average emission intensity. These environmental problems have inevitably hindered the improvement of regional EWP and further retarded sustainable development in the YREB. Therefore, research is needed to inform policy makers about how to improve and balance the relationship among economy, society and the ecological environment and achieve the high-quality coordinated and integrated development in the YREB.

Existing research demonstrates that there is a geographic gradient differentiation pattern in industrial ecological efficiency, green economic efficiency and factor agglomeration capacity across the YREB [8,9,10], but few scholars have probed the temporal and spatial differentiation of the EWP at an urban scale from the visual angle of human well-being. While ecological welfare studies are abundant, most focus on the national and provincial scales in analyzing spatial and temporal patterns. In reasoning the influencing factors of regional ecological welfare, fewer studies have employed spatial panel econometric models to investigate spatial effects [11]. In fact, the differences in EWP across different regions are not only constrained by their own resource endowments, but also are jointly affected by multiple effects of external factors, such as factor flow, technology diffusion and differential policy implementation. These effects can often be explained by means of a spatial panel Durbin convergence model in which the spatial interaction effect of “local-neighbor” is considered [12].

This research develops an all-round EWP index to assess the YREB’s EWP at a city scale. It attempts to reveal the spatial differentiation in the EWP and explore its driving forces using a spatial panel model. This study contributes to the theorization of comprehensive evaluation of regional EWP by incorporating three dimensions of economy, society and ecological environment into building a comprehensive EWP index and its associated analytical indicators. The developed EWP index improves the current index evaluation system for regional sustainable development evaluation. Methodologically, this research utilizes the spatial convergence model for estimating the spatio-temporal convergence of EWP across the cities in the YREB. The proposed model enables one to investigate the spatial effects of driving forces and their spatial interaction effects of “local-neighbors” in configuring the spatial patterns of regional ecological welfare, which are rarely examined in the existing studies. The empirical evidence generated in this research will contribute to figure out the relationship between regional economy, society and environment, and also provide a scientific basis for formulating policies aiming for the high-quality coordinated regional development.

## 2. Literature Review

Due to pressing problems associated with environmental degradation, economic growth and social welfare fluctuation, there is a growing interest in understanding how to promote sustainable regional development that simultaneously considers economy, society and ecological environment. Numerous studies have been published on EWP. Three broad categories of relevant studies can be identified: (1) the measurement of EWP; (2) the regional difference analysis of EWP; (3) the analysis of influential factors on the spatial–temporal changes of EWP. 

Daly first introduced the concept of EWP when evaluating the sustainable development in various countries, but it was not widely used due to its difficulty in quantification and low practicability of the proposed indices [13]. Over time, the idea of EWP was reconceptualized to include economic dimensions, which led to the development of comprehensive welfare indicators. Among these indicators, the human development index (HDI) is one of the widely adopted indicators in evaluating EWP. As a comprehensive index, the HDI takes into account the basic essentials of human development by considering life expectancy at birth, education status and income level in its quantification. It embraces both economic benefits on account of national revenue and non-economic benefits on account of social choices. This composite approach using a limited number of variables is convenient for calculation and analysis [14,15]. However, HDI still has two shortcomings. First, HDI focuses too much on economic growth and fails to consider the influence of environmental factors on human welfare. The index does not pay enough attention to ecology and ignores information about environmental sustainability. Second, HDI only considers the “output” of human welfare but fails to include the cost invested for this purpose [16]. To address the deficiencies of the HDI, some scholars such as Yew, Abdallah and Caillon et al. employed the ratio method to build the ecological performance evaluation index system [17,18,19], where the numerator is the social welfare indicators that are excluded by the HDI, and the denominator measures the consumption of ecological resources. However, none of the above methods measure the level of regional EWP comprehensively. 

The measurement method of EWP has gradually evolved into a more acceptable efficiency measurement method based on the input-output analysis framework [20,21,22]. There are two categories of efficiency measurement methods. The first is the non-parametric approach of Data Envelopment Analysis (DEA), and the second is the parametric approach of Stochastic Frontier Analysis (SFA). Although the influence of interference items is considered in SFA, the functional relationship between input and output needs to be assumed in advance, which has a certain degree of subjectivity [23]. The DEA model has become one of the most widely used models in the literature due to its advantages of requiring fewer indicator variables and retaining completely the original indicator information. DEA models include CRS (Constant Return to Scale), VRS (Variable Return to Scale), SBM (Slack-Based-Measure), three-stage DEA, Malmquist index, and the super-efficiency DEA model. Among them, the SBM model is widely used in the literature, because it can better deal with the negative environmental pollution output in the consumption of resource elements [24,25]. 

**Figure 1:**
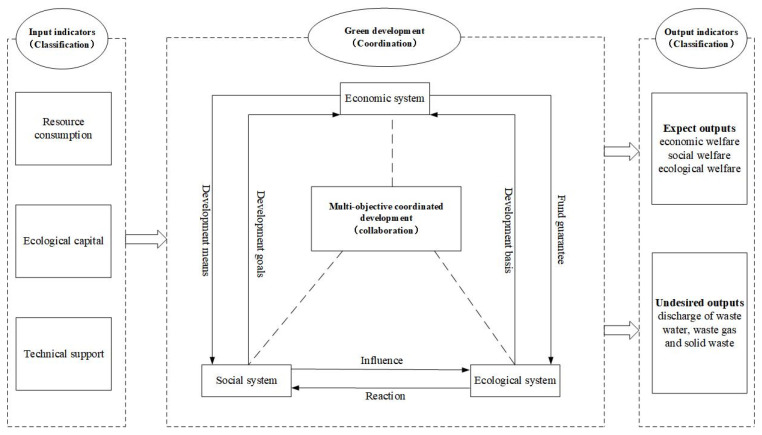
“Classification–Coordination–Collaboration” Analysis Framework of EWP.

The current input–output based evaluation index system and measurement methods of regional EWP are however limited by indicator selection, data collection and analysis methods. First, the existing researches only consider resource consumption as an input indicator, ignoring the capital and technological factors that transform resource consumption into outputs. Second, the current studies tend only to include industrial wastes as undesirable outputs for measurement, but exclude household waste discharge, PM_2.5_ and other indicators which are intimately linked to human well-being. Third, the current measure of people’s living standards only considers a single economic dimension. Indicators on education, medical care and health need to be integrated into the existing input–output analysis framework. To address these deficiencies, this paper proposes a “3C” (Classification, Coordination, Collaboration) analysis framework for the EWP (Figure 1) based on the “3C” theorization for sustainable development in the literature [26]. The framework provides a basis for developing a comprehensive evaluation index system and selecting appropriate evaluation indicators and measurements. In the framework, input indicators include resource consumption, ecological capital and technological capital, and output indicators consist of desired outputs and non-desired outputs. The goals of achieving ecological protection and green development are multi-dimensional and need to be coordinated. A multi-objective collaborative regional development model in the evaluation index system needs to be established for promoting the overall realization of the high-quality sustainable development of economy, society and ecological environment.

The literature on the regional differences in the EWP has so far focused on the national and provincial scales, and paid less attention to the city scale [27]. For example, Zhang et al. compared and analyzed the comprehensive evaluation values of EWP in 82 countries. It was found that developed countries and G20 countries performed relatively worse [28]. Long used cross-sectional data of 42 countries to conduct a horizontal international comparison, and found that the EWP was the highest in OECD countries, followed by G20 countries, and the lowest in BRICS [29]. Xu et al. measured the EWP of 30 provinces and cities in mainland China from 2005 to 2014. They found that the EWP generally showed an “east > central > west” gradient, and revealed the pattern of high–high or low–low agglomeration in the local space, indicating that China’s EWP presented a certain degree of spatial heterogeneity and spatial autocorrelation [30]. Fang and Xiao, Wang et al. calculated the provincial-level data in different periods of 2005–2016 and 1997–2018 based on a super-SBM model of undesired outputs, which also supports the conclusion found by Xu et al. [31,32]. Most of the above studies attempt to understand the spatial differences in EWP at the national, provincial and other macro scales. Little is known about how such differences manifest across various cities and other mesoscale units. In fact, the conclusions on EWP differences and causal mechanisms drawn from the macro scale analysis may not be applicable to the mesoscale, making it difficult to provide an effective reference for the policy-making of prefecture-level cities at the mesoscale. The YREB, which spans eastern and western China and connects its north and south, is a significant strategic support belt for national development in the new era, and it is also an ecologically sensitive area. However, the existing studies pay little attention to the temporal and spatial pattern of EWP at the urban scale in the belt. This paper, therefore, attempts to supply gaps in literature and will investigate the regional differentiation and dynamic trends in the YREB at a city scale. 

Regarding the research on the influencing factors of EWP, scholars have mainly investigated the effects of economic growth, industrial structure, urbanization, technological progress, and foreign direct investment [33]. However, the knowledge about the role of causal factors in influencing EWP patterns remains to be limited. For example, the relationship between economic growth and the EWP has been studied extensively in the literature, but findings seem inconclusive. Common used the 2001 cross-sectional data to find that the richer countries have lower EWP, and the EWP is negatively associated with economic growth [34]. Dietz et al. conducted a cross-country panel data analysis and reached a similar conclusion [35]. However, Jorgenson et al. used transnational panel data in their study and found that economic growth may not cause the EWP to decline [36]. The effects of other influencing factors on the EWP seem mixed. For example, Xiao and Zhang discovered that urbanization and industrialization have significant negative spatial spillover effects on the EWP [37]. Ma et al. found that urban growth, industrial structure and government investment have a promoting effect on the EWP, while foreign direct investment has an inhibitory effect [38].

While many factors may affect the EWP, the effects of influencing factors may vary significantly. The mixed findings on the effects of influencing factors may result from the data and analytical methods employed in various studies. The existing analysis methods investigating the influencing factors of EWP include cross section regression, panel Tobit, and LMDI. These analytical methods are often employed to explore the relations between EWP and social variables and are not suitable for detecting spatial effects [10]. In fact, the estimation results that ignore spatial effects are often biased [39]. Further, most of these methods are considered static models as they are incapable of revealing temporal trends of the associations between EWP and causal factors. The spatial convergence model (SCM) is used on the basis of spatial angles for investigating the differentiated causes of the associations among different regions and their convergence trends [40,41,42]. Therefore, this research builds a spatial Durbin convergence model which incorporates the spatial impacts. The study helps reveal the dynamic driving forces of regional differentiation in the EWP of the YREB.

## 3. Methods and Data 

### 3.1. Methods

#### 3.1.1. Measurement of EWP

Charns first presented the Data Envelopment Analysis (DEA). It is used to measure the efficiency of a multi-input–output DMU [43]. The initial DEA model is based on either radial or angular measurement. The radial method requires that inputs and outputs must change in the same proportion when evaluating efficiency, so it cannot address the slackness in inputs and outputs and the resulting measurement errors. The angular method requires that the evaluation of efficiency must be based on the perspective of input or output, and cannot give consideration to both. To solve the above problems, Tone presented a non-radial and non-angular DEA model on the basis of slackness variables, namely the SBM (Slack-Based Measure) model [44]. However, plural DMUs will be effective simultaneously when using the SBM model to measure efficiency, so it is unable to evaluate and rank DMUs effectively. The super-efficiency SBM model is an improvement on the SBM model, which can further evaluate the effective units (the efficiency value is 1), so as to obtain more accurate measurement results. The model is as follows:

(1) $$\begin{array}{l} \rho_{se}=\min\frac{1-\frac{1}{m}\sum_{i=1}^{m}\frac{s_{i}^{-}}{x_{ik}}}{1+\frac{1}{q_{1}+q_{2}}(\sum_{r=1}^{q_{1}}\frac{s_{r}^{g}}{y_{rk}^{g}}+\sum_{r=1}^{q_{2}}\frac{s_{r}^{b}}{y_{rk}^{b}})}\\ s.t.\left\{ \begin{array}{l} x_{k}=X\lambda +s^{-}\\ y_{k}^{g}=Y^{g}\lambda -s^{g}\\ y_{k}^{b}=Y^{b}\lambda +s^{b}\\ \lambda \geq 0,s^{-}\geq 0,s^{g}\geq 0,s^{b}\geq 0 \end{array}\right. \end{array}$$

where, *x_k_*, *y^g^_k_* and *y^b^_k_* are the input, desirable output and undesirable output vectors of the DMUs, respectively. *X_k_*, *Y^g^* and *Y^b^* are input, desirable output and undesirable output matrices. *s^−^, s^g^, s^b^* are the slackness of inputs, desirable outputs and non-desirable outputs. *λ* is the weight column vector. *ρ*_se_ is the measured value of efficiency. When 0 < *ρ*_se_ < 1, there is redundancy in the DMUs, and the efficiency can be raised by optimizing the input structure. When 1 ≤ *ρ_se_*, the DMU is valid.

#### 3.1.2. Dagum Gini Coefficient

The Gini coefficient and subgroup decomposition method is presented by Dagum [45]. Compared with the traditional imbalance measurement indexes, it can decompose the indexes into regional differences, inter-regional differences and hypervariable density, and solve the problems of subsample distribution, overlap and regional differences, so that it is easier to obtain indexes with economic implications. The calculation formula is as follows:

(2) $$G=\frac{\sum_{e=1}^{k}\sum_{f=1}^{k}\sum_{i=1}^{n_{e}}\sum_{j=1}^{n_{f}}\left|x_{ei}-x_{fj}\right|}{2n^{2}\bar{x}}$$

where, *G* denotes the Dagum Gini coefficient, $\bar{x}$ denotes the average value of the EWP in the YREB, *n* denotes the total number of research units in the study area (108 in this paper), *k* denotes the number of areas, *n_e_* (*n_f_*) denotes the number of research units in area *e* (*f*), and *x_ei_* (*x_fj_*) is the EWP of *no.i* (*j*) research unit in the region *e (f*). *G_ee_* and *G_ef_* are shown as follows:

(3) $$G_{ee}=\frac{\sum_{i=1}^{n_{e}}\sum_{j=1}^{n_{f}}\left|x_{ei}-x_{fj}\right|}{2n_{e}{}^{2}\bar{x}}$$

(4) $$G_{ef}=\frac{\sum_{i=1}^{n_{e}}\sum_{j=1}^{n_{f}}\left|x_{ei}-x_{fj}\right|}{n_{e}n_{f}(\bar{x_{e}}-\bar{x_{f}})}$$

Based on the decomposition of Dagum Gini coefficient, the total Gini coefficient is broken down into regional difference (*G_rd_*), inter-regional difference (*G_ird_*) and hypervariable density (*G_t_*), and *G* = *G_rd_* + *G_nb_* + *G_t_*. The expression is as follows:

(5) $$G_{rd}=\sum_{e=1}^{k}G_{ee}p_{e}s_{e}$$

(6) $$G_{ird}=\sum_{e=2}^{k}\sum_{f=1}^{e-1}D_{ef}G_{ef}(p_{e}s_{f}+p_{f}s_{e})$$

(7) $$G_{t}=\sum_{e=2}^{k}\sum_{f=1}^{e-1}(1-D_{ef})G_{ef}(p_{e}s_{f}+p_{f}s_{e})$$

where, $p_{e}=n_{e}/n$, $s_{e}=n_{e}{\bar{x}}_{e}/n\bar{x},\;$_._*D_ef_* is the relative influence of EWP between the regions *e* and *f*. *d_ef_* is the performance difference of ecological welfare in the region, representing the mathematical expectation of the sum of all samples ($x_{ei}-x_{fj}>0$) in the regions *e* and *f*, and *p_ef_* is the hypervariable first-order moment, representing the mathematical expectation of the sum of all samples ($x_{fj}-x_{ef}>0$) in the areas *e* and *f*. For details of each coefficient, refer to Stéphane and Patrick [46].

#### 3.1.3. Verification of Spatial Correlation

For investigating the spatial distribution of the EWP in the YREB comprehensively, the global spatial correlation index in ESDA was employed in this research. *Moran’s Index* was commonly employed for measuring the global spatial correlation. The results reflect the concentration level of spatial distribution of the EWP in the YREB, and embody the similarity of adjacent cities. The expression is as follows:

(8) $$Moran’s\;I=\frac{n\sum_{i=1}^{n}\sum_{j=1}^{n}w_{ij}(y_{i}-\bar{y})(y_{j}-\bar{y})}{\sum_{i=1}^{n}\sum_{j=1}^{n}w_{ij}\sum_{i=1}^{n}{\left(y_{i}-\bar{y}\right)}^{2}}$$

where *n* represents 108 cities in the YREB, *w_ij_* denotes the spatial weight metric, and *y* and $\bar{y}$ denote the EWP and its mean value, respectively. For exploring systematically the spatial relevance characteristics of the EWP, three spatial weight matrices were set and standardized based on the Queen Adjacent Matrix (*w_xy_*), Geographic Distance Matrix (*w_d_*) and Economic Distance Matrix (*w_e_*), as shown in Table 1.

#### 3.1.4. Spatial Panel Durbin Convergence Model

*β*-convergence was first used to analyze whether the economic development of a region will change with time in a steady state. In this paper, *β*-convergence means that regions with a low EWP are able to overtake regions with a high EWP, with a higher growth rate over time, and the gap between the two gradually narrows, eventually reaching the same stable level. *β*-convergence includes absolute and conditional *β*-convergence. Absolute *β*-convergence refers to all cities in YREB having identical economic and social characteristics, and the EWP of each prefecture-level city will tend to reach the equivalent level over time. The spatial dependence of different regions is not considered in the traditional standard *β*-convergence model, but the spatial panel Durbin convergence model can explain the influence of the spatial interaction effect of “local-neighbor” on the dependent variable, so this model was selected for investigating the convergence of EWP in the YREB. The expression is as follows:

(9) $$\begin{array}{ll} Ln(EWP_{i,t+1}/EWP_{i,t})=\alpha & +\rho \sum_{j=1}^{n}W_{ij}Ln(EWP_{i,t+1}/EWP_{i,t})+\beta LnEWP_{i,t}\\ & +\lambda \sum_{j=1}^{n}W_{ij}LnEWP_{i,t}+\delta_{t}+\mu_{i,t} \end{array}$$

where, *EWP_i,t_*, and *EWP_i,t+_*_1_ are the EWP of the city *i* in year *t* and *t* + 1 respectively, *W_ij_* refers to the spatial weight matrix, and *ρ* refers to the spatial autoregression coefficient, indicating the impact of the growth rate of EWP in adjacent cities on the local city. *λ* refers to the impact of EWP in adjacent cities on the local city. *δ_t_* denotes time fixed effect. *μ_i,t_* denotes a random perturbation term. *β* is the convergence coefficient. If *β* is significantly negative, it means that regions with a lower EWP have faster growth rates than the regions with higher EWP, and there is *β*-convergence. On the contrary, if it is positive, there is no convergence.

Secondly, the conditional *β*-convergence of EWP is not just affected by the initial factor endowment of each region, but also by the surrounding external social–economic factors, which will lead to differences in its steady-state values. Therefore, the spatial lag term and the influence factors of surrounding areas (WX) were selected to construct the spatial panel Durbin model. The convergence expression is:

(10) $$\begin{array}{c} Ln(EWP_{i,t+1}/EWP_{i,t})=\alpha +\rho \sum_{j=1}^{n}W_{ij}Ln(EWP_{i,t+1}/EWP_{i,t})+\beta LnEWP_{i,t}+\sum_{k=1}^{n}\theta_{k}X_{i,t}\\ +\lambda \sum_{k=1}^{n}W_{ij}EWP_{i,t}+\varphi \sum_{k=1}^{n}W_{ij}X_{i,t}+\delta_{t}+\mu_{i,t} \end{array}$$

In this expression, *θ_k_* denotes the coefficient of the influencing factors and *φ* refers to the impact of influencing factors in adjacent cities on the local city. Other coefficients are the same as above. 

### 3.2. Establishment of Indicator System

#### 3.2.1. Establishment of Indicator System

According to the “3C” analysis framework of EWP, resource consumption, ecological capital and technological capital are considered to be the ecological inputs with reference to ecological welfare input–output indicator selection proposed by Zhang et al. and Li et al. [47,48,49]. In addition to the traditional resource consumption indicators, ecological capital and technology input are also included and measured by urban environmental infrastructure investment, per capita science and technology and education expenditure. It includes a multi-dimensional comprehensive appraising indicator system of both well-being outputs and non-well-being outputs. Among them, the non-well-being output is an indicator of environmental pollution. In addition to the three industrial wastes commonly used by scholars [50], it also includes domestic waste removal and transportation volume and PM_2.5_ particles that are closely related to the life quality of residents. In terms of desirable outputs, relevant indicators reflecting economic benefits and education level [51] were selected, and environmental, medical and health aspects were also taken into consideration. They are represented by the park land area per capita and the number of doctors per 10,000 inhabitants, respectively. The comprehensive appraising indicator system of the EWP along with multi-source data is listed as follows.

This research employs the statistical data from *China Urban Statistical Yearbook*, *China Urban Construction Statistical Yearbook* and statistical yearbooks of provinces and cities from 2009 to 2019. The data of PM_2.5_ in the undesirable outputs comes from the grid data of PM_2.5_ concentrations released by the Social Economic Data and Application Center of Columbia University and obtained by satellite monitoring (https://sedac.ciesin.columbia.edu/data/set/sdei-global-annual-gwr-pm2-5-modis-misr-seawifs-aod-v4-gl-03 (accessed on 7 September 2022). The above data are described in Table 2.

#### 3.2.2. Selection of Influence Variables

When constructing the conditional *β* convergence model, this paper considers the influence of industrial structure, technological input, urbanization development level, environmental regulation, foreign capital utilization, government intervention and transportation accessibility on the convergence of EWP. Considering the availability of data, the specific indicators are as follows(see Table 3):

Industrial structure (*Ind*). Industrial upgrading and shifting service-oriented economy can effectively promote carbon emission reduction, improve environmental quality, and thus improve the EWP of residents [52]. The proportion of tertiary industry in GDP is used for measuring the industrial structure.

Technology input (*Tec*). Chen and Cheng argued that advances in technology are the main means for improving China’s extensive economic growth mode with high consumption and emissions [53]. Therefore, it is measured based on the sum of science and technology expenditure and education expenditure per capita.

*City*. Urbanization is one of the most influential human activities. On the one hand, urban growth will increase energy consumption, thereby reducing environmental quality [54]. On the other hand, the increasing level of urbanization contributes to the utilization efficiency of infrastructure and improves people’s living standards [55]. The proportion of the population in municipal districts of a prefectural city–region is used to characterize the urbanization process.

Environmental regulation (*Er*). On the one hand, environmental regulation may force some heavy pollution enterprises to withdraw from the market, thereby improving the environmental quality. Another aspect, environmental regulation could lead to higher production costs and environmental governance costs of enterprises, resulting in less productive investment and lower profits and consequently competitiveness of enterprises. Environmental regulation may thereby limit the improvement of enterprise production efficiency and affect economic development [56]. Comprehensive indicators such as domestic sewage treatment rate, industrial sulfur dioxide removal rate, industrial solid waste comprehensive utilization rate, and industrial dust removal rate are selected to illustrate the strength of environmental supervision.

Foreign direct investment (*Fdi*). The “pollution refuge” hypothesis believes that foreign direct investment will aggravate the environmental pollution of the host country under the conditions of loose environmental regulations [57]. The “pollution halo” hypothesis believes that the adoption of standardized management methods and advanced production technologies by foreign-funded enterprises can promote domestic enterprises to optimize their environmental management system through technology spillover effects, thus helping to improve the environmental quality of host countries [58]. The ratio of the actual amount of foreign investment used in a year to GDP is used for measuring foreign direct investment.

Government intervention (*Gov*). Government intervention determines the regional institutional environment to a large extent, thus affecting the efficiency of resource allocation and economic development [59]. The share of fiscal expenditure in GDP is used for measuring the governmental intervention.

Transportation accessibility (*Trans*). Convenient transportation can shorten the space and time distance between regions, promote the flow and exchange of regional resources, and thus improve the living standard of residents [60]. This research employs per capita urban road area for measuring the transportation convenience in each city. 

## 4. Spatial Variation of EWP in YREB

### 4.1. Spatial and Temporal Evolution of EWP 

**Figure 2:**
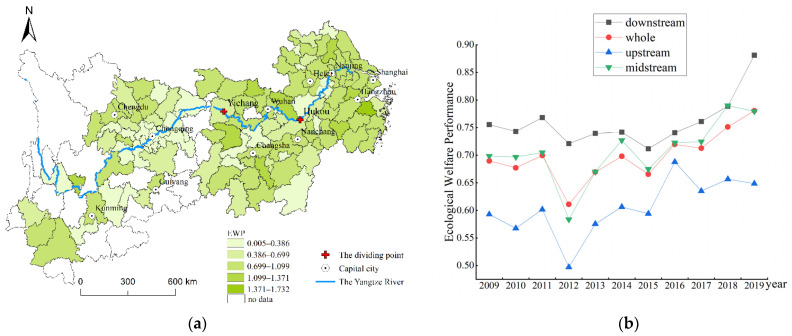
Distribution map of EWP in 2019 (**a**) and its dynamic changes (**b**) in the YREB.

Figure 2 displays the spatial distribution and temporal evolution of YREB’s EWP. The higher the EWP, the healthier urban development and greater people’s well-being will be. From the angle of spatial distribution, most of the high-value regions are concentrated in Jiangsu and Zhejiang in the downstream of Yangtze River, however, Wuhan, Changsha, Zhuzhou and Xiangtan in midstream, and provincial capital cities in the upstream are also associated with a high EWP, reflecting the high level of urban development in these regions and the effective improvement of residents’ well-being. Low-value areas are mostly located in non-capital cities in the upstream, including western Hubei and Hunan, and southern Jiangxi. Macroscopically, it presents a ladder-shaped distribution pattern of east-high and west-low, showing a gradual decline gradient. From the angle of temporal evolution, the EWP for each region in YREB shows a fluctuating but upward trend. The gap in EWP between different regions still exists, so further analysis of differences in the EWP between different regions in the YREB is essential. 

### 4.2. Regional Differences of EWP 

**Figure 3:**
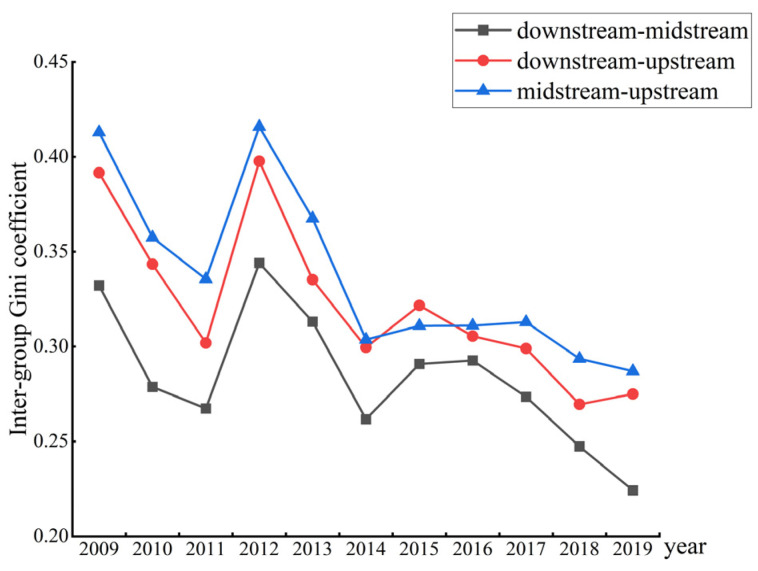
Regional differences of the EWP in YREB.

The Dagum Gini coefficient was adopted for measuring the regional variations about EWP in YREB (Figure 3). As a whole, the Gini coefficient of the YREB’s EWP is on the high side, but it fluctuates and declines in the sample period, indicating that there is a prominent geographic variation in the EWP among the prefecture level cities in the YREB, but the degree of spatial variation has been reduced. Specifically, the Gini coefficient in the upstream region of the YREB ranks first in all regions, because the upstream region of the YREB covers a wide area and has complex terrain. Furthermore, different cities have different resource endowments, varying degrees of industrialization, and different paces of economic development. The problem of unbalanced and insufficient high-quality development in the region is prominent. The EWP gap among cities in the midstream YREB is comparatively small and more balanced. The mean regional difference in the lower reaches of the YREB is the lowest, and it has maintained a lower level from 2009 to 2019, reflecting the positive impact of integrative and inclusive development in the YREB. Moreover, its internal central cities can generate positive spatial-spillover effects on surrounding cities, helping them improve their EWP.

### 4.3. Inter-Regional Differences of EWP 

**Figure 4:**
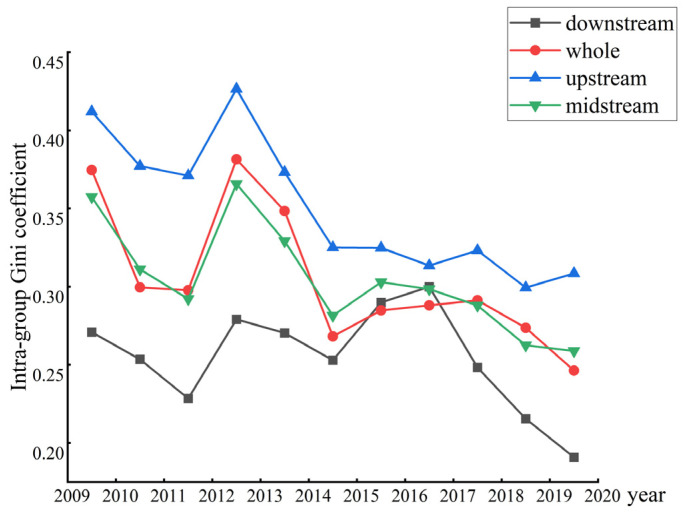
Inter-regional differences of the EWP in the YREB.

Figure 4 refers to the time-series evolution of the inter-regional variance of EWP between the downstream, midstream and upstream YREB during the sample observation period. From the overall trend, the Gini coefficient between paired regions is fluctuating and declining. Among them, the Gini coefficient between the midstream and the downstream is the smallest, and shows a further downward trend. The possible reason is that the midstream region may have benefitted from industrial diffusion out from the downstream region, optimized the industrial structure, and improved its own economic development, thus narrowing cross regional differences. In contrast, although the Gini coefficient between the downstream and upstream regions, and between the midstream and upstream regions, declined significantly during the sample observation period, it was still at a high level. It can be seen from the figure that the inter-regional difference in the EWP is the greatest between the downstream and upstream regions, followed by the difference between the midstream and upstream regions.

### 4.4. Overall Difference and Decomposition of EWP 

**Figure 5:**
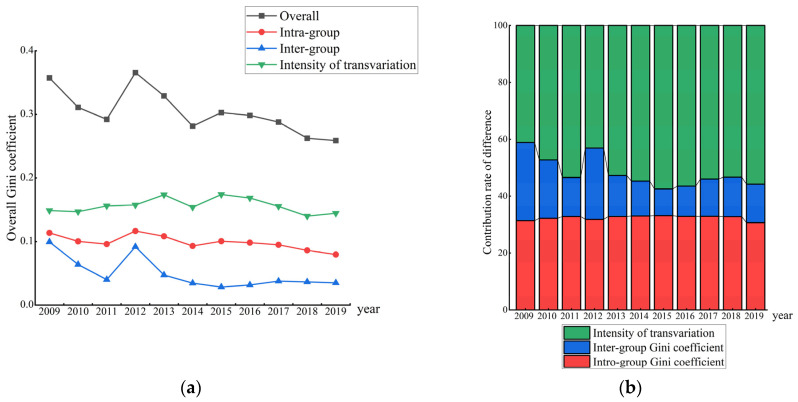
Spatial differentiation (**a**) and contribution rate (**b**) of EWP in the YREB.

Figure 5 shows the contribution rate of regional variance, inter-regional variance and hypervariable density in the EWP of the YREB. As shown in Figure 5 the whole variance of the EWP shows a fluctuating decline, which specifically exhibits an inverted “N” downward trend of decrease–increase–decrease, but the difference remains at a high level. In addition, the Gini coefficient between regions has decreased significantly, indicating that the gap in EWP among regions in the YREB has narrowed.

From the contribution rate of the variance (Figure 5b), the hypervariable density is the major source of the whole variance during the sample study period, and it is always higher than the regional and inter-regional variance, which denotes that the overlapping phenomenon between different regions of YREB is an important reason for the poor EWP. The hypervariable density reflects the spatial imbalance of the development of the EWP. While the developed regions have improved their EWP, the underdeveloped cities have “left behind” and failed to keep up with the pace of development. Therefore, we should not only pay attention to the underdeveloped regions and give them corresponding fiscal and tax subsidy policies, but also find out the reasons why the underdeveloped cities in the developed regions fail to develop, and take corresponding measures according to local conditions for narrowing the gap and promoting the coordinated development of the YREB.

## 5. Analysis on Spatial Differentiation and Drives of EWP in the YREB

### 5.1. Spatial Autocorrelation of EWP

*Moran’s I* of the EWP in the YREB during 2009–2019 was calculated by using ArcGIS software, and the results are presented in Table 4. It denotes that *Moran’s I* in all years has passed the significance test and is larger than zero, denoting that the spatial distribution of YREB‘s EWP during this period has an obvious spillover effect. The prefecture level cities with a high (or low) EWP are often adjacent and have spatial dependence, which may be mainly due to the regional economic integration. Each city is not an “island”; its EWP is no longer limited to its own development, and the influence of surrounding areas cannot be ignored.

### 5.2. Spatial Convergence Model Test

#### Model Selection

It can be seen from the previous analysis that the EWP has a strong spatial correlation. If the inherent spatial spillover effect is ignored when analyzing its spatial convergence trend, it is likely to obtain erroneous empirical results. In addition, not only the explained variable itself, but also the explanatory variable and error term will cause spatial autocorrelation. The spatial Durbin model (SPDM) could well recognize the spatial effects of different sources [61]. Thus, this research starts from the SPDM. First, LR tests and Wald tests were adopted for determining whether SPDM could degenerate to SAR or SEM. Results passed the 5% significance level test and the original hypothesis was rejected (see Table 5). The SPDM was employed to conduct empirical test. Then the existence form of spatial dependence was tested, and the results show that the spatial dependency of YREB’s EWP exists in the form of error. In addition, Hausman test results show that the log likelihood value of the time fixed effect model (1055.3) is greater than those of the space fixed effect model and the spatial–temporal fixed effect model (935.39, 900.2), so this research selected the time–fixed SPDM.

### 5.3. Absolute β-Convergence

Table 6 shows the estimation results of the absolute *β*-convergence model for YREB’s EWP under the three spatial matrices. It can be seen as follows. First, under the three matrix conditions, the *β*-convergence coefficients are all less than zero at the 0.01 significance level, indicating the gap has narrowed without considering the impact of other factors. Cities with relatively low EWP have a “catching-up effect” to the cities with a relatively high EWP, eventually converging to a steady state level. Second, although the significance is slightly different, *ρ* and *λ* are all positive, indicating that the change rate of the EWP of this city is affected by the change rate and the positive spatial spillover of other cities. Under the proximity matrix and geographical distance matrix, both are significantly positive, indicating that its change rate is more vulnerable to the impact of neighboring cities. Therefore, the local government shouldn’t only take into account its own development, but also think about the influence of neighboring cities when formulating relevant policies to improve the EWP of each city.

### 5.4. Conditional β-Convergence Analysis

Table 7 denotes the conditional *β*-convergence analysis for the EWP of 108 prefecture level cities in YREB. First, the results show there is significant conditional *β*-convergence in the YREB. Second, the conditional *β*-convergence rate has increased compared to absolute *β*-convergence, indicating the convergent rate is driven by the industrial structure, city, environmental regulation, foreign investment, transportation accessibility and other factors.

According to the regression results, this research obtains several discoveries. First, the overall spatial spillover effect of the YREB has not changed, and both *ρ* and *λ* are positive, indicating that the change rate of EWP of each city is subject to the impact of neighboring cities. Second, from the perspective of economics, the convergence of each control variable on the EWP of the YREB is significantly different. Specifically, the adjustment of industrial structure (*Ind*) and technological innovation (*Tec*) have a generally positive effect, which can significantly promote the overall EWP to converge to a high value, but the spatial spillover effects of both are significantly negative, possibly because the development of the tertiary industry, especially the modern service industry, can improve the efficiency of resource utilization, and the improvement of technological level reduces the pollution emissions of unit products, thus promoting the improvement of EWP. However, on the one hand, the transformation and upgrading of the industrial structure of the surrounding cities will enhance their competitiveness; on the other hand, they will transfer their outdated production capacity to other regions, thus preventing them from converging to high values. Secondly, the acceleration of urbanization will not only promote regional growth, but also improve the infrastructure construction, health care, employment, education and income of residents in the surrounding areas, so to facilitate EWP convergence to a higher value. Thirdly, environmental regulation (*Er*) inhibits the convergence of EWP, and its spatial spillover effect is significantly negative. The reason may be that the current environmental regulation is still in the “passive governance“ stage. With the deepening of pollution, the governance will be strengthened, so the overall level of environmental regulation shows a certain inhibition. Fourthly, foreign direct investment (*Fdi*) has significantly inhibited the convergence of the YREB’s EWP, but its spatial spillover effect is positive. The possible reason is that there are huge differences among cities in the YREB. Some developed cities have attracted foreign capital early, and the role of foreign capital has gone beyond the stage of advanced management concepts and technologies to accelerate the convergence of EWP to a high value. However, for some underdeveloped regions, the advanced technology and management level brought by foreign investment can continuously spread from developed regions with the flow of personnel and resource elements, thus promoting the EWP of underdeveloped regions to converge to a high value, so its spatial spillover effect is positive. Fifthly, transportation accessibility (*Trans*) has a generally positive effect on the overall EWP in the YREB. The improvement of transportation will help guide the efficient distribution and flow of various elements and expand the living space of residents. Under the effect of agglomeration and scale, social security and public service facilities will develop in a convenient and reasonable direction, and this sharing will greatly enhance people’s sense of happiness. Finally, the impact of governmental intervention (*Gov*) on the convergence of the YREB’s EWP is not significant, which does not mean that the governmental intervention has no impact on the EWP. Rather, government intervention cannot promote the EWP in the YREB to converge to high or low values.

### 5.5. Robustness Test

For testing the authority of the regression outcomes, this research replaces the different spatial matrices. From the regression outcomes in Table 8, the YREB’s EWP has a significant conditional convergence trend under the different spatial matrices. At the same time, the significance level of other control variables on the convergence of EWP is not much different from the results in Table 8, which shows that the spatial econometric model that was designed is robust.

### 5.6. Heterogeneity Analysis

The YREB has a vast territory, with different conditions of urban development in the upstream, midstream and downstream, and the problem of unbalanced and insufficient regional development is also very prominent. At the same time, there are obvious differences in economic development, regional advantages, factor endowments and industrial structures among each region, which makes the influencing factors of EWP heterogeneous across different city–region in the YREB. Therefore, this paper further investigated the convergence of EWP in the upstream, midstream and downstream of the Yangtze River. The estimated results are shown in Table 9. Several points need to be noted. First, significant conditional *β*-convergences are presented in the YREB as well as in its lower, middle and upper reaches. However, there are different rates of convergence. The difference in spatial differentiation of the EWP is manifested through different convergence rates. Cities in the midstream of the Yangtze River have the fastest convergence rate, followed by upstream cities, and downstream cities are the slowest. Second, the spatial effects of different regions in YREB are different. *ρ* and *λ* in the midstream and upstream of the YREB are both negative, showing a negative spatial spillover effect, because the development of cities in midstream and upstream is extremely uneven, and most of those with a high EWP are concentrated in the developed provincial capital and central cities where most of the regional education, medical care, science and technology resources have been accumulated, and the siphon effect is significant. *ρ* and *λ* in the lower reaches of the YREB are positive, showing a positive spillover effect of “high interdependence”, because the integrated regional development is actively promoted in YREB’s downstream, especially the Yangtze River Delta. Talents, resources and technology in developed cities with a high EWP can be circulated to the developing cities, thus driving their EWP to converge to high values. Third, the spatial differentiation in the upstream, midstream and downstream of YREB is manifested through different convergence rates, and the differences in convergence rates in different regions are mainly driven by the combined effects of industrial structure, technology input, urbanization, environmental regulation, foreign direct investment, transportation accessibility and other factors. Different driving factors have significant heterogeneous impacts on the EWP’s convergence (Table 9). For instance, technology input inhibits the convergence of the EWP in midstream regions, the city has significant negative effects on the EWP in upstream regions, and transportation accessibility has significant heterogeneous effects on EWP. Obviously, the regional heterogeneity is caused by the different resource endowment, the social and economic development and the characteristics of the main stages of development in different regions.

## 6. Discussion and Conclusions

Based on the “3C” analysis framework of “classification, coordination and collaboration”, this research developed a comprehensive appraising indicator system of inputs and outputs that considers economy, environment and society simultaneously for measuring the efficiency of regional EWP. Multi-source data were integrated and the super-efficiency SBM model was used for measuring EWP of 108 cities in the YREB from 2009 to 2019. Then, the Dagum Gini coefficient decomposition method was used for exploring the integral and regional variations in EWP, and the SPDM of convergence was employed to explore driving factors of their spatial differentiation. 

The study identifies that there are gradient differences in the EWP of the YREB from the perspective of regional differences, and this difference has gradually narrowed over time, confirming the finding by Bian et al. that there are regional variations in China’s EWP [27]. In addition, it reveals the existence of regional differences in the EWP, and finds that hypervariable density has always played a primary role in the integral difference of the YREB’s EWP, indicating that underdeveloped cities in developed regions have lagged behind. From the angle of inter-regional differences, Wang et al. studied the differences in EWP based on provincial panel data, and found that the internal variations of the EWP in the eastern region were the maximum [32]. This paper offers new insight into regional differences at the urban scale. EWP differences in the upper reaches are the largest among all regions, reflecting the prominent imbalance of the development of their internal EWP. The difference of the lower reaches is the smallest, which indicates that the steady progress of the integration process in the Yangtze River Delta will help narrow the gap in EWP across the cities.

Second, the spatial-clustering features of the YREB’s EWP have been analyzed through ESDA. During the sample study period, the *Global Moran’s I* in all years was greater than zero. It is concluded that the spatial distribution of the YREB’s EWP has obvious spillover effects, and its distribution shows “H-H” and “L-L” agglomeration features. This finding confirms the spatial distribution characteristics of EWP observed at the national scale by Bian et al. [33], indicating a cross-scale similarity in spatial clustering of the EWP. 

Third, some scholars have investigated the evolution of resource allocation efficiency, green total factor productivity and carbon emission rate from the perspective of spatial convergence [40,41,42], but few scholars use spatial convergence models to study the differences in EWP. By constructing a spatial panel Durbin convergence model, this study proposes and verifies the difference in the convergence rate of the EWP and the main influencing factors in the upstream, midstream and downstream of the YREB. It also supplements and improves the research contents of EWP from the angle of spatio-temporal evolution, offering a novel method to relevant scholars to continue to engage in this research. 

Fourth, in explaining the influence of causal factors on EWP, the extant studies often employ traditional non-spatial econometric paradigms and the spatial auto-correlation is often ignored. In fact, the evident spatial agglomeration and spatially correlated distribution of EWP require us to consider the spatial spillover effect in analyzing its changes and driving forces; otherwise, it will lead to biased analytical results. Ma et al. [38] pointed out that industrial structure, urbanization and government regulation are conducive to improving the EWP, but the use of foreign capital will have a negative effect. The findings of this study confirm that these factors not only influence the EWP but also how it converges over time. In particular, its spatial effect cannot be ignored in modeling spatial and temporal characteristics of the EWP. Specifically, our study indicates industrial structure and technology investment can promote the convergence of the EWP to a high value, but their spatial dispersion effects play an opposite role. Further, foreign capital and environmental regulation can have a significant restraining effect on the convergence of EWP, but the spatial effect of neighboring foreign capital is positive and the spatial effect of environmental regulation of neighboring cities is negative. These results provide an enhanced understanding of how spatial interactive effects of the influencing factors on EWP may behave differently from their non-spatial effects, indicating the necessity of using spatial models in future research.

Fifth, the heterogeneity analysis is another highlight of this paper. The existing research on EWP convergence tends to treat the study area as a unified homogeneous whole and its internal differences are often ignored [21,62]. This study reveals that the impact size and direction of influencing factors vary across different regions within the study area of the YREB. The heterogeneity of the YREB is identified by dividing it into three parts of upstream, midstream and downstream. The results confirm that the direct and spatial effects of different influencing factors are heterogeneous, and this heterogeneity is also evident in the EWP convergence speed. The cities in the midstream of the Yangtze River converge fastest, followed by the cities in the upstream, and the cities in the downstream are the slowest. From the angle of heterogeneity, this paper offers a novel angle to the study of the EWP.

The present research still has some limitations. First, objective indicators were selected based on the availability of data in the selection of indicators related to EWP. This study does not include subjective well-being indicators that should be considered in future studies. Second, the convergence of EWP was the outcome of the interactive impacts of many factors. This study only considered the direct impact of the influencing factors. The indirect and interactive effects of these factors need to be further studied. Third, while this study reveals the association of the influencing factors with the convergence of EWP, future studies may need to explore the causal mechanism of these factors using a system dynamics model.

## 7. Policy Implications

There are several policy implications from the findings of this research. First, the regional imbalance of urban development in the YREB should be focused on. It is necessary to strengthen the spreading effects from central cities to surrounding cities, reduce the backwash effect, and stop the spatially polarizing development pattern in the YREB. *Global Moran’s I* shows that the distribution of EWP is characterized by a remarkable regional interdependence. Therefore, cities or regions in geographical proximity and complementary development modes should be encouraged to formulate coordinated development plans and strive to realize the free flow of production factors, product outputs, technologies, and labor forces within the region. Because the spatial spillover effect cannot be ignored, the local government must consider the impact of surrounding areas, especially in the developed regions. In formulating relevant policies, the impact on the surrounding under-developed areas must be considered. Furthermore, it is required to establish a long-term cooperation for coordinated development strategies between the developed and the developing regions in order to promote coordinated sustainable regional development. Second, due to the differences in the convergence rate of the EWP in the upstream, midstream and downstream of the YREB and the different influencing factors, the local government should propose and follow a targeted development path according to the local characteristics of each region. As far as the lower reaches are concerned, social inequity, income gaps and other aspects of human and ecological wellbeing should be focused on achieving coordinated regional sustainable development. For the middle reaches, it is urgent to speed up the upgrading of industrial structure, eliminate backwards production capacity, increase attraction to talent, provide corresponding support policies, and focus on people’s living standards in the development process. For the upper reaches, it is necessary to further expand foreign direct investment, increase infrastructure construction, and make full use of their own ecological advantages to transform them into ecological and economic advantages.

## Figures and Tables

**Table 1:** Spatial weight matrix types.

Type of Spatial Weight Matrix	*w_xy_*		*w_xy_*	
	Value	Condition	Value	Condition
Adjacency	1	Cities *x* and *y* are adjacent	0	Cities *x* and *y* are not adjacent
Geographical distance	1/*d_xy_^2^*	*x* ≠ *y*	0	*x* = *y*
Economic distance	$\frac{1/\left|g_{x}-g_{y}\right|}{\sum_{x,y}^{n}1/\left|g_{x}-g_{y}\right|}$	*x* ≠ *y*	0	*x* = *y*

Note: *d_xy_* denotes the distance between city *x* and city *y*; *g_x_*, *g_y_* represent the per capita GDP of city *x* and city *y* respectively, *n* denotes the total number of cities.

**Table 2:** Appraising indicator system of the EWP in the YREB.

Category	First Indexes	Secondary Indexes	Tertiary Indexes	Unit
Input	Resource consumption	Land consumption	Per capita construction land	m^2^/person m^2^/person
		Energy consumption	Per capita household electricity consumption	kWh/person
		Water resource consumption	Total water supply per capita	ton/person
	Ecological capital	Ecological input	Per capita investment in urban environmental infrastructure	RMB/person
	Technical support	Technology input	Per capita expenditure on science & technology and education	RMB/person
Output	Undesirable output	Solid waste discharge	Per capita urban domestic waste removal volume	ton/person
		Wastewater discharge	Industrial wastewater	ton/person
		Exhaust gas emission	Industrial sulfur dioxide	ton/person
			Industrial fumes	ton/person
			PM_2.5_	ug/m^3^
	Desirable output	Economic development	GDP per capita	RMB/person
			Total retail sales of consumer goods per capita	RMB/person
			Per capita public financial income	RMB/person
		Environmentally friendly	Park land per capita	m^2^/person
		Social development	Urban road per capita	m^2^/person
			Number of ordinary university populations Per 10,000 persons	Person
			Number of doctors per 10,000 inhabitants	Person

**Table 3:** Control variables and their descriptive statistics.

Variable	Mean	Std	Min	Max	Obs
*Ind*	1.8333	0.2264	0.278	4.617	1080
*Tec*	6.912	0.7133	0.9942	9.692	1080
*City*	2.238	0.2667	1.993	2.482	1080
*Er*	4.237	0.1466	0.388	7.254	1080
*Fdi*	0.663	1.2529	0.123	3.747	1080
*Gov*	3.773	0.4099	2.028	2.554	1080
*Trans*	1.506	0.6016	0.209	9.932	1080

**Table 4:** *Moran’s I* of overall EWP during 2009~2019.

Year	*Moran’s I*	Z Value	Year	*Moran’s I*	Z Value
2009	0.1234 **	0.0330	2015	0.1388 **	0.0177
2010	0.1413 **	0.0108	2016	0.1317 **	0.0119
2011	0.2008 ***	0.0086	2017	0.1276 **	0.0434
2012	0.1426 **	0.0150	2018	0.1407 **	0.0142
2013	0.1292 **	0.0265	2019	0.1512 ***	0.0099
2014	0.1848 ***	0.0032	Average	0.1468 **	0.0125

Note: ***, ** refer to significant levels of 0.01 and 0.05, respectively.

**Table 5:** Spatial panel econometric model test.

Target	Statistic	Value	*p*-Value
Selection of econometric model	LR lag	15.9137	0.0436
	Wald lag	16.1865	0.0398
	LR error	14.6661	0.0660
	Wald error	14.5368	0.0688
Form of existence of spatial dependence	LM test no lag	22.4830	0.0000
	Robust LM test no lag	0.3692	0.543
	LM test no error	27.8227	0.0000
	Robust LM test no error	5.7089	0.017

**Table 6:** Absolute *β*-convergence estimation results.

Variable	Adjacency Matrix	Geographic Distance Matrix	Economic Distance Matrix
*β*	−0.4852 *** (−18.792)	−0.4836 *** (−18.719)	−0.4720 *** (−18.2812)
*ρ*	0.1974 *** (3.3456)	0.5876 *** (2.9389)	0.0406 (0.5469)
*λ*	0.1409 *** (2.942)	0.1429 *** (2.6487)	0.1449 *** (2.4889)
*R^2^*	0.3145	0.3160	0.3082
*log-l*	1032.01	1034.18	1036.20

Note: *** refer to significant levels of 0.01, with t-values in parentheses.

**Table 7:** Conditional *β* convergence estimation results under adjacent space weight matrixes.

Variables	Adjacency Matrix	
	Coefficient	WX
*β*	−0.5261 *** (−20.177)	
*ρ*	0.1032 ** (3.0755)	
*λ*	0.1898 ** (3.0179)	
*Ind*	0.1032 *** (3.0755)	−0.0902 * (−1.7568)
*Tec*	0.1631 *** (3.2080)	−0.1449 * (−1.8970)
*City*	0.4151 (1.2641)	0.5779 ** (1.9682)
*Er*	−0.2415 * (−1.7010)	−0.4951 ** (-2.3089)
*Fdi*	−0.0488 *** (−2.6750)	0.0568 * (1.8575)
*Gov*	−0.0110 (−0.8621)	−0.6486 (-1.5417)
*Trans*	0.0791 ** (2.2307)	−0.1074 (0.1187)
*R^2^*	0.3863	
*log-l*	1042.30	

Note: ***, ** and * refer to significant levels of 0.01, 0.05 and 0.1 respectively, with t-values in parentheses. WX indicating the impact of the X of neighboring cities on the city. W is the spatial weight matrix.

**Table 8:** Conditional *β* convergence estimation results of two spatial weight matrices.

Variables	Geographical Distance Matrix		Economic Distance Matrix	
	Coefficient	WX	Coefficient	WX
*β*	−0.5266 *** (−20.031)		−0.5250 *** (−19.783)	
*ρ*	0.3152 (1.2156)		0.0730 (0.9580)	
*λ*	0.2569 ** (1.9740)		0.1299 ** (2.2303)	
*Ind*	0.0948 *** (2.864)	−0.3551 ** (−2.6397)	0.0503 * (1.7212)	0.0266 (0.3431)
*Tec*	0.1591 *** (3.2727)	−0.1915 (−0.4910)	0.0706 (1.4398)	0.0583 (0.3856)
*City*	0.4834 * (1.4779)	0.5742 * (1.7698)	0.7687 ** (2.3119)	−0.1443 (−1.1624)
*Er*	−0.2696 * (−1.8806)	−0.3810 *** (−3.4266)	−0.3136 ** (−2.202)	0.6297 ** (−2.052)
*Fdi*	−0.0429 ** (−2.3512)	0.2602 *** (2.4189)	−0.0587 *** (−3.5853)	0.1508 *** (2.7869)
*Gov*	−0.0313 (−0.0313)	−0.4286 ** (−2.2128)	0.0026 (0.0359)	0.0625 (1.1451)
*Trans*	0.0835 ** (2.3679)	−0.2521 (−0.8224)	0.0556 (1.6779)	−0.0609 (−0.6203)
*R^2^*	0.3475		0.3082	
*log-l*	1004.07		1036.20	

Note: ***, ** and * refer to significant levels of 0.01, 0.05 and 0.1 respectively, with t-values in parentheses.

**Table 9:** Analysis results of heterogeneity of the EWP based on regional differences.

Variable	Lower Reaches	Middle Reaches	Upper Reaches
*β*	−0.6270 *** (−14.0731)	−0.6675 *** (−13.6775)	−0.6620 *** (−13.3374)
*ρ*	0.0675 (0.6293)	−0.2728 ** (−1.9397)	−0.2046 * (0.0278)
*λ*	0.2069 *** (0.0034)	−0.1019 (−1.1964)	0.2409 * (0.0871)
*Ind*	0.1609 *** (2.6953)	0.0765 (1.1173)	0.1522 *** (2.6894)
*Tec*	0.0489 (0.4019)	−0.1367 ** (2.0258)	0.2022 * (1.7917)
*City*	1.0770 ** (2.086)	0.6146 (0.9631)	−0.7820 ** (−2.0899)
*Er*	−0.0927 (−0.2469)	−0.1225 (−0.2646)	−0.1145 (−0.6531)
*Fdi*	0.0172 (0.5318)	−0.0782 * (−1.7589)	0.0572 ** (−2.1325)
*Gov*	0.0131 (0.1127)	0.0495 (1.8959)	0.0956 (0.8252)
*Trans*	−0.0539 ** (−1.9309)	0.2037 *** (3.0708)	0.0193 (0.7030)
*R^2^*	0.3688	0.4779	0.4143
*log-l*	380.60	327.96	302.13

Note: Note: ***, ** and * refer to significant levels of 0.01, 0.05 and 0.1 respectively, with t-values in parentheses. Due to layout limitations, regression coefficients for WX are not reported in the table.

## Data Availability

The data presented in this study are available from the corresponding author.
